# Impact of the natural female reproductive aging on the rat serum lipidome

**DOI:** 10.1042/CS20255841

**Published:** 2025-09-18

**Authors:** Julio Baudin, Anna Antolín, Salvador Fernández-Arroyo, Antoni Del Pino, Francisca Mulero, Francesc Puiggròs, Lluís Arola, Antoni Caimari

**Affiliations:** 1 Technological Unit of Nutrition and Health, Eurecat - Centre Tecnològic de Catalunya, Reus, 43204, Spain; 2 Nutrigenomics Research Group, Department of Biochemistry and Biotechnology, Universitat Rovira i Virgili, Tarragona, 43007, Spain; 3 Centre for Omic Sciences, Joint Unit Eurecat–Universitat Rovira i Virgili, Unique Scientific and Technical Infrastructure (ICTS), Eurecat - Centre Tecnològic de Catalunya, Reus, 43204, Spain; 4 Molecular Imaging Unit, Spanish National Cancer Research Centre (CNIO), Madrid, Spain.; 5 Biotechnology Area, Eurecat - Centre Tecnològic de Catalunya, Reus, 43204, Spain

**Keywords:** early biomarkers, hormonal changes, lipidomics, menopausal transition, metabolomics, ovarian function, reproductive aging

## Abstract

Perimenopause is a transitional phase leading to female reproductive senescence, which can cause vasomotor symptoms and increase the risk of osteoporosis, obesity, and metabolic-related disturbances in middle-aged and older women. Nevertheless, little is known regarding the underlying mechanisms linked to menopausal transition, which could be of great value in designing new interventions addressed to improve the health of both perimenopausal and postmenopausal women. We used an ovarian-intact middle-aged model of rats resembling the characteristics of human perimenopause and applied liquid and gas chromatography quadrupole time-of-flight mass spectrometry approaches for the determination of polar and lipid-related metabolites to identify characteristic circulating signatures across perimenopause. The gradual loss of regularity in the estrous cycle occurring during the natural transition to reproductive senescence was associated with altered circulating levels of estradiol, progesterone, and luteinizing hormone (LH) and, in rats that were in an acyclic state, with ovary atrophy and with a lack of stromal luteinization and corpus luteum. These results were accompanied by progressively significant changes in the 144 lipid-related metabolites detected in serum as the estrous cycles were losing regularity. Furthermore, we identified 18 lipid-related metabolites—including 9 phosphatidylcholines, 4 lysophosphatidylcholines, 2 phosphatidylethanolamines, cholesterol ester (18:2), 5α-androstane-3,17-diol, and 17,18-dihydroxyarachidonic acid—that already changed with the transition from a regular to an irregular estrous cycle and anticipated the changes in blood progesterone, LH, and cholesterol levels that occurred in acyclic rats. These metabolites could be used as a potential multivariate biomarker of early perimenopause. The translational applicability of these findings deserves further research.

## Introduction

Perimenopause is a transitional phase leading to menopause, which can extend over several years with varying onset, duration, and completion times [1,2] . During this transition, women experience a variety of physical and psychological symptoms that could be directly or indirectly linked to changes in sex hormone secretion due to a dysregulation of the hypothalamic–pituitary–ovarian (HPO) axis, more extended periods between consecutive menstrual cycles, gradual decline in ovarian follicular function, and other physiological changes [2–4]. The appearance of the first symptoms related to the menopausal transition is usually vasomotor, such as hot flashes, night sweats, sleep alterations, migraines, and affective disorders. However, the duration and intensity of these symptoms can vary greatly among women, ethnicity, and age of first experience [5,6]. In contrast, postmenopausal symptoms tend to be more detectable due to increased health risk factors associated with complete estrogen deficiency, including osteoporosis, cardiovascular disease, stroke, insulin resistance, diabetes, metabolic syndrome (MetS), and ovarian cancer [7–10]. Thus, the variability in the vasomotor symptoms and the lack of longitudinal studies tracking women through the perimenopausal transition makes it difficult to establish a clear understanding of perimenopausal symptoms and their underlying mechanisms linked to menopausal transition.

Preclinical studies with rodents have demonstrated that rats provide a well-established model for natural reproductive aging. The examination of vaginal smears (cytology) and vaginal histology is commonly used methods for the identification of the different stages of the estrous cycle and assessing the ovarian function during the reproductive senescence [11–14]. The estrous cycle characterization is crucial, as oocytes have no regenerative ability, and any ovarian lesions may be directly linked to impairment in female reproductive aging. Ovulation in rats occurs at a regular interval of four or five days in young-mature adult female rats (sexual maturity at around 8 weeks of age) [15,16]. In middle-aged female rats, approximately seven to eight months of age [15,17,18], they proceed through sequential reproductive stages. During this period, they progressively transition from regular estrous cycles to irregular patterns, eventually becoming acyclic, characterized by persistent estrus. After the cessation of ovulation and entry into persistent estrus, there is still some maturation of follicles, followed by atresia without ovulation, culminating in complete follicular depletion, arriving when the rats are about 20 months old to the final stage of reproductive senescence in old rats, which is named persistent anestrus [11,15,19]. This progression in rat female reproductive aging mirrors the transitions seen in women from premenopausal, perimenopausal, and postmenopausal stages [17,20,21].

Despite these similarities in reproductive aging between female rats and women, there has been limited exploration of rodents' natural transition to acyclicity to gain insight into adjacent mechanisms of reproductive senescence progression. This is partly due to the more common use of ovariectomized models, which help replicate rapid hormone depletion and evaluate the postmenopausal stage but may miss underlying mechanism changes that initiate and perpetuate the dysregulation of reproductive physiology in middle age [21,22].

Different studies have identified molecular neuroendocrine changes in the hypothalamus during the natural progression from regular reproductive cycles to acyclicity in middle-aged female rats, comparable with the perimenopausal progression in women [21]. These changes were associated with altered genes related to sex hormone feedback in HPO functions and the DNA methylation of genes required for hormone signaling, as well as melatonin and circadian pathways during the different stages of natural reproductive aging in female Sprague-Dawley rats [18,23]. From a translational perspective, it would be of great value to use biological samples obtained in an easy and minimally invasive way to detect changes across perimenopause to settle the bases for designing and implementing strategies and interventions at the early stages of natural reproductive senescence aimed at preserving the endocrine and metabolic function in women. Recent studies in women have revealed an association between reproductive senescence in the postmenopausal stage and alterations in the circulating lipidome. Post-menopausal women tended to exhibit accelerated patterns of lipidome aging compared with their pre-menopausal counterparts of the same biological age [24,25]. However, the cause–effect relationship between lipidomic changes and reproductive senescence remains unclear. To the best of our knowledge, no studies based on metabolomic and lipidomic approaches have identified circulating signatures characteristic of perimenopause. We hypothesized here that changes in circulating metabolite patterns already occur during the menopausal transition and, consequently, appear before reaching an advanced stage of menopause.

The objectives of this study were (1) to identify novel circulating metabolites influenced by natural reproductive senescence in female rats, (2) to elucidate further changes across perimenopause in the metabolome and lipidome to gain insight into the mechanisms underlying natural reproductive aging, (3) to identify potential non-invasive early biomarkers of perimenopause during the transition from regular estrous cycles to irregular cycles. For this purpose, we deeply characterized a rat model resembling the characteristics of human perimenopause and used both liquid and gas chromatography quadrupole time-of-flight mass spectrometry methods for the determination of polar and lipid-related metabolites to identify characteristic circulating signatures across perimenopause. We reported changes in lipid-related metabolites with the appearance of irregular estrous cycles, regardless of age, which could pave the way to design and implement potential therapies in a very early stage of female reproductive aging to slow down or ameliorate the health risk factors that can appear with menopause.

## Materials and methods

### Animal care

All procedures related to animal experimentation were approved by the Animal Ethics Committee of the Technological Unit of Nutrition and Health of Eurecat (Reus, Spain) and the Generalitat de Catalunya (protocol code 11223). The study complied with the ARRIVE guidelines, followed the ‘Principles of Laboratory Animal Care’, and was carried out in accordance with the EU Directive 2010/63/EU for animal experiments. Twelve young (3.5 months old, sexually naïve) and sixty middle-aged (8 months old) female Sprague Dawley rats (INOTIV, Horst, Netherlands) were used at the beginning of the study. Upon arrival and throughout the entire duration of the study, all rats were housed in pairs at the animal facility of Eurecat (Reus, Spain), maintained at 22°C with a 12 h light/dark cycle (lights on at 9:00 am), and provided *ad libitum* access to food and water. All animals were fed a standard chow diet, renewed daily (Teklad Global 14% Protein Rodent Diet 2014, Harlan, Barcelona, Spain), and their body weight and food intake were recorded weekly. The caloric composition of the diet (2.9 kcal/g) was 20% protein, 13% fat, and 67% carbohydrates.

A daily assessment of the estrous cycling of female rats was conducted using the procedure described by McLean *et al*. [26]. To ensure the rats were less stressed or more accustomed to handling, one week prior to the study’s commencement, rats were handled for 5 minutes per day before monitoring estrous cyclicity through daily vaginal lavage with ultrapure distilled water. The cycle status was evaluated daily via cytology of uterine cells obtained from vaginal lavage at 11 am. Vaginal smears were morphologically characterized based on the four stages of the cycle: estrus, metestrus, diestrus, and proestrus [16,26]. Animals and their cycle regularity were categorized as regular, irregular, or acyclic based on the last 14 days of their smears. To ensure a more accurate interpretation of vaginal cytology and estrous cycle stages, consecutive daily samples were collected and analyzed collectively after one week. This approach aimed to minimize differences in cell morphology among rats and reduce the likelihood of capturing transition stages in the estrous cycle during sample collection. Regular animals exhibited three consecutive 4- to 5-day cycles, irregular animals showed two consecutive cycles that were 6 days or longer (extended diestrus phase), and acyclic animals displayed 14 continuous days in persistent estrus (constant/alternative estrus or proestrus phases). Rats that exhibited persistent estrus during the initial 14 days were excluded from the study. This decision focused on evaluating biomarkers or early changes in the menopausal transition, such as the duration of acyclicity in these rats before the survey, which was unknown. Based on the age and the regularity in the last 14 days of estrous cycle status, rats were sacrificed by beheading under anesthesia (pentobarbital sodium, 60 mg/kg body weight) after 4 h of diurnal fasting at proestrus (regular and irregular groups) or persistent estrus (acyclic group) and later assigned to one of the following four categories: young rats with regular cycling (Reg-Yng; *n* = 10, ~ 5 months), middle-aged rats with regular cycling (Reg-Ma; *n* = 7, ~ 10 months), middle-aged rats with irregular cycling (Irreg-Ma; *n* = 8, ~ 10 months), and middle-aged rats with persistent estrus (Acyc-Ma; *n* = 8, ~ 10 months). Rats that did not meet the cytological criteria for each group were excluded from analyses for this study. Blood was collected by beheading, and serum was obtained by centrifugation and stored at -80°C until analysis. The liver, cecum, kidneys, left ovary, gastrocnemius, soleus muscle, right femur, and the white adipose tissue depots (mesenteric -MWAT- and periovarian -POWAT- white adipose tissue depots) were rapidly removed, weighed, frozen in liquid nitrogen, and stored at −70°C until further analysis. The right ovary was removed from attached tissues such as perigonadal white adipose tissue and uterine horns, weighted, and fixed in 4 vol % neutral buffered formalin until ovarian cytology and histologic analyses. The left femur was also collected, immersed in 70% ethanol, and stored at room temperature until carrying out a three-dimensional microcomputed tomography (microCT) analysis.

### Homeostasis model assessment-estimated insulin resistance and revised quantitative insulin sensitivity check index analyses

The homeostasis model assessment-estimated insulin resistance (HOMA-IR) was calculated using the following formula: (fasting glucose level [mmol/l] × fasting insulin level [µU/ml]/22.5) [27]. Insulin sensitivity was evaluated by the revised quantitative insulin sensitivity check index (R-QUICKI) using the following formula: 1/[log insulin (µU/ml) + log glucose (mg/dl) + log FFA (mmol/l)] [28].

### Serum analysis

Enzymatic colorimetric kits were used to determine serum total cholesterol (TC) (995280/QCA, Barcelona, Spain), triglycerides (TG) (992330/QCA, Barcelona, Spain), glucose (992330/QCA, Barcelona, Spain) and non-esterified free fatty acids (NEFAs) (WAKO, Neuss, Germany). Circulating insulin levels were measured using a rat/mouse ELISA kit (10–1250-01/MERCODIA, Uppsala, Sweden), and C-reactive protein (CRP) was quantified using the ERCRP ELISA kit (INVITROGEN, Massachusetts, U.S.A.). Serum leptin levels were determined with a rat ELISA kit (EZRL-83K/Millipore, Barcelona, Spain), and serum adiponectin levels were quantified by a Rat Total Adiponectin/Acrp30 Quantikine ELISA Kit (RRP300/R&D systems, Minnesota, U.S.A.).

Serum estradiol (17β-E2) concentrations were quantified by Laboratori Echevarne (Barcelona, Spain) using the Atellica IM eE2 assay, a competitive chemiluminescent immunoassay based on an acridinium ester-labeled monoclonal sheep antibody. Briefly, endogenous 17β-E2 was released from its binding proteins by a releasing agent. A labeled antibody was then added to bind to the available hormone. A capture conjugate coupled to magnetic latex particles was also introduced, competing with endogenous 17β-E2 for binding. After washing, acid and base were added to initiate a chemiluminescent reaction. The amount of hormone present in the sample is inversely proportional to the relative light units (RLUs) detected by the instrument.

To quantify the remaining circulating female sex hormones—progesterone (P), luteinizing hormone (LH), and follicle-stimulating hormone (FSH)—the following ELISA kits were used: progesterone (CSB-E07282r/CUSABIO, Houston, U.S.A.), LH (CSB-E12654r/CUSABIO, Houston, U.S.A.), and FSH (CEA830Ra/Cloud-Clone Corp., Houston, U.S.A.).

### Cytological and histological analyses

The excised right ovary from each rat was immediately preserved in buffered formalin (4 % formaldehyde, 4 gr/L NaH2PO4, 6.5 gr/L Na2HPO4; pH 6.8), sectioned at a thickness of 5 µm, and subsequently stained with hematoxylin & eosin (H & E). Ovarian images (magnification 15×) were captured using a microscope (SMZ745T; Nikon, Tokyo, Japan) coupled with a digital sight camera (DS-Ri1, Nikon). Ovarian examinations were performed according to the criteria of previous reports [12,13,15,29]. To avoid any bias in the analysis, the study employed a double-blind design, preventing the researchers from viewing any data from the rats during the histopathological analysis. A detailed microscopic examination of the prepared ovarian and the criteria for assessment were established through a double correlation by professional histopathologists (Eldine Patologia SLP, Tarragona, Spain). The ovarian histopathological examination included an evaluation of follicular development (changes in the number of primordial, primary, secondary, and antral follicles development, presence/absence of corpus luteum, changes in development, regression in the number of corpus luteum) as previously described [19,29,30] in addition to any lesions, such as the presence/absence of ovarian atrophy. Features of the ovaries throughout the reproductive aging period are described in detail in the Results section.

### Microcomputed tomography analyses

Bone composition of the left femur was determined post-mortem in fixed samples by three-dimensional microCT analysis, as was previously described [31]. The bone mineral content (BMC) and bone mineral density (BMD) values were quantified from microCT scans using the GE MicroView software v2.2.

### Sample preparation and analysis of polar metabolites by GC-EI-qTOF-MS

Metabolites were extracted from serum using a hydroalcoholic solution. 100 µl of serum sample was added to 400 µl of a solution of 80% methanol containing the internal standards, succinic-D4 acid, myristic acid-D27, glucose-13C6, and L-Methionine-(carboxy-13C, methyl-D3) (Sigma-Aldrich, St. Louis, MO) and labeled amino acid mix standards (Cambridge Isotope Laboratories, Massachusetts, U.S.A.). Samples were vortexed and centrifuged for 5 minutes at 15000 rpm and 4°C. Supernatants (450 µl) were transferred to a new tube and evaporated in a SpeedVac at 45°C to dryness. Afterward, samples were reconstituted with 30 µl of methoxyamine (40 mg/ml) in pyridine and incubated for 90 min at 37°C. Finally, samples were silylated with 45 µl of MSTFA + 1% TMCS at room temperature for 60 min.

A semi-targeted analysis of the serum polar metabolites was performed using a Gas Chromatography-Quadrupole Time-of-Flight (GC-qTOF) 7200 equipped with an electron impact (EI) source (Agilent Technologies, Palo Alto, California, U.S.A.). The chromatographic separation was performed with helium (purity > 99.999%) as carrier gas at a constant flow of 1.1 ml/min. The ramp temperature to separate derivatized polar compounds was as follows: initial GC oven temperature was 60°C; 1 min after injection, the GC oven temperature was increased by 10 °C/min to 320°C and held for 10 min. In the inlet, 1 µl of samples was injected with split mode 1:20 at 250°C. Detection was achieved using mass spectrometry (MS) in electron impact (70 eV) mode and full-scan monitoring mode (50–600 *m/z*) with an acquisition rate of 5 spectra/s. The ion source and quadrupole temperatures were set at 250°C and 200°C, respectively. Identification of metabolites was performed using commercial standards and by matching their EI mass spectrum and retention time to the metabolomic Fiehn library (from Agilent Technologies, Palo Alto, California, U.S.A.), which contains more than 1,400 metabolites. The identification was carried out using exact mass data with an accepted mass error of 20 ppm. The internal standards used were succinic-D4 acid, myristic acid-D27, glucose-13C6, and L-methionine-(carboxy-13C, methyl-D3) (Sigma Aldrich) and labeled amino acid mix standards (Cambridge Isotope Laboratories). After the putative identification of metabolites, these were semi-quantified in terms of internal standard response ratio.

### Sample preparation and analysis of polar and non-polar lipids by UHPLC-ESI-qTOF-MS

Electrospray ionization (ESI- and ESI+) was employed to cover all ranges of metabolites for the semi-targeted analysis of the serum lipids. For methanol extraction (ESI-), serum samples were thawed at 4 °C. 50 µl of serum was mixed with 200 μl of 100% methanol and a set of labeled internal standards: cholic acid, chenodeoxycholic acid, deoxycholic acid, hyodeoxycholic acid, lithocholic acid, ursodeoxycholic acid, taurocholic acid, taurochenodeoxycholic acid, taurodeoxycholic acid, taurolithocholic acid, glycocholic acid, glycochenodeoxycholic acid, glycodeoxycholic acid, tauroursodeoxycholic acid, decanoic acid c10:0, dodecanoic acid c12:0, palmitic acid c16:0, stearic acid c18:0, oleic acid c18:1, linoleic acid c18:2, linolenic acid c18:3, arachidic acid c20:0, arachidonic acid c20:4, t4, dehydroisoandrosterone 3-sulfate (DHEAS), estrone sulfate, corticosterone, myristic-d27, cortisol-d4, 4-hydroxy-3-methoxymandelic acid-d3, 3-hydroxytetradecanoic acid-2,2,3,4,4-d5, cholic acid-d4, all of them were purchased to Sigma Aldrich (St. Louis, MO); LysoPE(18:1), LysoPC(20:0), LysoPC(16:0), LysoPC(18:0), LysoPC(18:1), LysoPE(16:0), LysoPC(18:1)-d7, all of them were purchased from Avanti Polar Lipids (Alabaster, U.S.A.); 12(13)-DiHOME, 9(10)-DiHOME, 9(s)-HODE, 13(s)-HODE, 12(13)-EpOME, 9(10)-EpOME, thromboxane B2, 15(s)-HETE, 12(s)-HETE, AA-d8, oxy deuterated linoleic acid oxylipins, deuterated primary COX and LOX, all of them were purchased from Cayman Chemical (Michigan, U.S.A.); and 3-(3-hydroxy-4-methoxyphenyl)propionic acid d3, Taurocholic Acid-d5, both were purchased to Toronto Research Chemicals, Toronto, Canada. The mixture was vortexed and centrifuged for 10 min at 15,000 rpm at 4 °C. The supernatant was evaporated to dryness using a SpeedVac concentrator, reconstituted with 50 μl of methanol, and analyzed by an Ultra-High-Performance Liquid Chromatography analysis, UHPLC-(-ESI)-qTOF-MS. For hydrophobic lipids (ESI+), liquid-liquid extraction with chloroform:methanol (2:1) based on the Folch procedure was performed. Briefly, 20 µl of serum was mixed with 20 µl of 0.8% NaCl and vortexed. Then, 200 µl of chloroform:methanol (2:1) was added, and samples were vortexed and centrifuged at 15000 rpm at 4 °C for 10 minutes. The lower phase was recovered, evaporated to dryness, reconstituted with 100 µl of methanol:methyl-tert-butyl ether (9:1), and analyzed by UHPLC-(+ESI)-qTOF-MS using a set of labeled lipid internal standards (SPLASH, purchased to Avanti Polar Lipids (Alabaster, U.S.A.): LysoPC(18:1)-d7, PC(33:1)-d7, SM(36:2)-d9, DG(33:1)-d7, TG(48:1)-d7 and ChoE(18:1)-d7.

For the semi-targeted analysis of the serum lipids by methanol extraction (ESI-) and Folch extraction (ESI+), both methods utilized an Ultra-High-Performance Liquid Chromatograph (UHPLC) 1290 Infinity II Series coupled with a Q-TOF iFunnel 6550 mass spectrometer (Agilent Technologies, Palo Alto, California, U.S.A.). Chromatographic separation employed different analytical columns: a CORTECS C18 Column (1.6 μm, 2.1 × 150 mm) from Waters for the methanol extraction method (ESI-) and a Kinetex 2.6 μm EVO-C18 column (100 Å, 100 × 2.1 mm) from Phenomenex for the Folch extraction method (ESI+). For the ESI- methodology, chromatographic separation was performed with a mobile phase of 100% water with 0.05% acetic acid for phase A and 100% acetonitrile for phase B at a 0.4 ml/min flow rate. The column temperature was maintained at 40°C, and the injection volume was 2 μl. For the ESI + methodology, the chromatographic method involved elution with a mobile phase consisting of water (phase A), methanol (phase B), 2-propanol (phase C), and 200 mM ammonium formate and 2% formic acid (phase D) at a 0.6 ml/min flow rate. The stationary phase was a Kinetex EVO C18 column (2.6 μm, 2.1 mm × 100 mm), thermostated at 60°C, enabling the sequential elution of hydrophobic lipids such as lysophospholipids, sphingomyelins, phospholipids, diglycerides, triglycerides, and cholesteryl esters, among others. The chromatographic separation for ESI + and ESI- was performed using the gradients conditions and source parameters detailed in online supplementary tables 1; 2. Identification of lipid metabolites was carried out using commercial standards, along with accurate mass matching (error < 20 ppm) and tandem mass spectrometry data, when available, against the Agilent METLIN-PCDL and LipidMaps libraries. Additionally, chromatographic behavior of pure standards for each lipid class and relevant bibliographic information were used to support putative.

### Multivariate and univariate statistical analyses in metabolomics and lipidomics datasets

Statistical multivariate analyses were conducted using serum metabolomic and lipidomic profiles obtained from middle-aged rat groups (Reg-Ma, Irreg-Ma, and Acyc-Ma) to assess the impact of the transition in female reproductive aging on the circulating metabolome. These analyses were performed using RStudio v.2024.04.1 for data visualization. Principal component analysis (PCA) was initially conducted to explore overall variability and grouping trends among the three groups based on 403 identified lipid metabolites. Subsequently, partial least-squares discriminant analysis (PLS-DA) was carried out to assess global metabolic changes across the menopausal transition groups. The classification performance of the PLS-DA model, constructed with all identified lipid metabolites, was evaluated using receiver operating characteristic (ROC) curve analysis. Its robustness and reproducibility were validated through a permutation test with 999 iterations and a 5-fold cross-validation method, with a random seed of 1 set before the analysis.

To identify early multivariate biomarkers for diagnosing the menopausal transition, the top 40 discriminant lipids were selected based on their Variable Importance in Projection (VIP) scores (>1) from the initial PLS-DA model. Subsequently, a refined PLS-DA model was constructed using only these top 40 metabolites. The performance of this new model was assessed using cumulative R² and Q² values, and its predictive accuracy was confirmed through ROC curve analysis. A ROC value greater than 0.7 among groups was considered indicative of a good discriminative capacity of the model.

Analysis of variance (ANOVA) was employed to identify significant differences in the normalized abundances of the filtered features. To account for multiple comparisons and control false discovery rates (FDR), significance was determined using the Benjamini-Hochberg correction with an adjusted p-value (p adj) < 0.05. Fold change values between groups for the significant lipids were calculated within RStudio. All statistically significant results at FDR < 0.05 were utilized for further analyses. Statistical multivariate analyses were conducted using hierarchical clustering analysis (HCA or heatmap), Bonferroni-adjusted Pearson correlation matrix (p adj. < 0.05), boxplots, as well as PCA. For PCAs and boxplots among middle-aged rat groups, we utilized the R/Bioconductor package ggplot2 v3.4.4 [32]. For PLS-DA, VIP score values, permutation test, 5-fold cross-validation, and ROC curves, the R/Bioconductor package mixOmics v.6.26.0 was used. For the calculation of accumulative Q² and R² values in the OPLS analysis, we used the package ropls v1.34. The Bonferroni-adjusted Pearson correlation matrix was generated using the R/Bioconductor package corrplot v.092. The heatmaps were generated using the R/Bioconductor package ComplexHeatmap v2.16.0 [33], with the Euclidean distance metric selected. The study utilized various biological databases such as HMDB, PubChem, and LIPID MAPS, along with scientific literature included in PubMed, to gain insight into the biological function of the metabolites that were identified as statistically significant among groups and with the top 40 discriminant lipids detected. The lipids were referenced according to the HMDB nomenclature recommendations.

### Statistical analyses

Statistical analyses were performed to detect differences among the four groups in biometric, histological, and biochemical parameters with IBM SPSS Statistics 28.0 (SPSS, IBM Corp. Armonk, New York, U.S.A.). Grubbs' test was used to detect outliers, which were discarded for further analysis. The normality assumption was determined using the Kolmogorov-Smirnov test, and the homoscedasticity among groups was evaluated using Levene’s test. When one or both conditions were not accomplished, data were transformed to base-10 logarithms to obtain a normal distribution and/or similar variances before statistical testing. One-way ANOVA followed by Duncan’s *post hoc* test (p-value ≤ 0.05) was used to assess differences among the four groups in most of the variables analyzed in the present study. Welch’s test, followed by Games‒Howell’s post hoc test, was used when homoscedasticity was not assumed. The Kruskal‒Wallis test, followed by the Mann‒Whitney U post hoc test, was used as the nonparametric version of one-way ANOVA when the data did not follow a normal distribution. Differences in ovarian atrophy and stromal luteinization were detected using the Chi-squared test. Linear relationships between key variables were tested using Pearson’s correlation coefficients. Student’s t-test was also used for some single statistical comparisons to detect residual differences. Data are presented as SEM (*n* = 7–10). The level of statistical significance was set at bilateral 5%.

## Results

### Monitorization and characterization of the different stages of natural reproductive senescence in female rats

The cycling status of 3.5- and 8-month-old female Sprague-Dawley rats was assessed longitudinally over a period of approximately 2 months in which the rats were stratified into groups according to the stage of ovarian senescence following the classification of the human perimenopause-menopause transition as per Stages of Reproductive Aging Workshop (STRAW). At the end of this process, all the rats included in the Reg-Yng group (*n* = 10), which were included to distinguish chronological aging, displayed regular cycling (showing cycles of 4–5 days), whereas 23 middle-aged rats were selected to be included in the Reg-Ma group (*n* = 7, displaying cycles of 4–5 days), in the Irreg-Ma group (*n* = 8, showing two consecutive cycles that were 6 days or longer) and in the Acyc-Ma group (*n* = 8, displaying 14 continuous days in persistent estrus) (Table 1). At the end of the study, the Reg-Yng animals were approximately 5 months old, whereas the middle-aged rats were about 10 months old, with no significant differences in age among the three middle-aged groups (Table 1).

**Table 1:** Biometric parameters and serum parameters in female rats during natural reproductive aging.

	Reg-Yng (*n* = 10)	Reg-Ma (*n* = 7)	Irreg-Ma (*n* = 8)	Acyc-Ma (*n* = 8)
Initial age (days ≈ months)	106 ≈ 3.5	247 ≈ 8	247 ≈ 8	247 ≈ 8
Final age (days ≈ months)	157 ≈ 5	295 ≈ 10	294 ≈ 10	293 ≈ 10
Cycling status	Regular	Regular	Irregular	Acyclic
Biometric parameters				
Initial body weight (g)	238 ± 3^a^	273 ± 7^b^	281 ± 11^b^	263 ± 4^b^
Final body weight (g)	268 ± 6^ab^	282 ± 6^a^	288 ± 11^a^	257 ± 5^b^
Tissue weights (%)				
Uterine horns (%)	0.166 ± 0.020^a^	0.160 ± 0.018^a^	0.143 ± 0.013^a^	0.232 ± 0.014^b^
POWAT (%)	1.59 ± 0.19^ab^	2.13 ± 0.23^a^	2.17 ± 0.28^a^	1.48 ± 0.15^b^
Liver (%)	2.67 ± 0.09^a^	2.26 ± 0.04^b^	2.48 ± 0.09^a^	2.53 ± 0.05^a^
Caecum (%)	1.26 ± 0.12	1.11 ± 0.06	1.38 ± 0.13	1.15 ± 0.09
MWAT (%)	0.94 ± 0.11	1.20 ± 0.09	1.13 ± 0.16	0.93 ± 0.10
Gastrocnemius (%)	0.714 ± 0.013	0.660 ± 0.037	0.678 ± 0.021	0.698 ± 0.024
Soleus (%)	0.044 ± 0.001	0.043 ± 0.004	0.039 ± 0.003	0.045 ± 0.003
Serum parameters				
Glucose (mmol/l)	6.28 ± 0.22	6.22 ± 0.20	6.00 ± 0.22	6.35 ± 0.14
Insulin (pmol/l)	19.6 ± 1.5	20.6 ± 0.9	23.3 ± 2.4	27.8 ± 3.6
NEFAs (mmol/l)	0.88 ± 0.04	1.04 ± 0.06	0.93 ± 0.05	0.95 ± 0.07
R-QUICKI	0.039 ± 0.002	0.036 ± 0.001	0.034 ± 0.002	0.030 ± 0.002
HOMA-IR	5.53 ± 0.57	5.73 ± 0.33	5.58 ± 0.49	7.95 ± 1.21
TG (mmol/l)	0.96 ± 0.06	0.90 ± 0.10	1.10 ± 0.10	1.21 ± 0.13
TC (mmol/l)	4.02 ± 0.21^ab^	3.45 ± 0.22^a^	4.00 ± 0.22^ab^	4.45 ± 0.22^b^
Adiponectin (µg/ml)	15.5 ± 1.7^ab^	17.2 ± 1.3^a^	15.9 ± 1.5^ab^	11.2 ± 0.5^b^
Leptin (ng/ml)	3.60 ± 0.68^a^	8.71 ± 1.62^b^	6.45 ± 1.18^b^	4.77 ± 0.61^ab^
Adiponectin/leptin ratio	5.08 ± 0.69^a^	2.65 ± 0.63^b^	2.81 ± 0.36^b^	2.55 ± 0.30^b^
Leptin/adiponectin ratio	0.235 ± 0.032^a^	0.545 ± 0.131^b^	0.351 ± 0.046^b^	0.428 ± 0.048^b^
CRP (µg/ml)	613 ± 99	452 ± 62	605 ± 76	742 ± 100

Data are given as the mean ± SEM (n = 7-10). Relative tissue weights (%) were calculated according to the formula (100* tissue weight/body weight) and were expressed as a percentage of body weight. The concentrations of serum parameters were determined at the end of the experiment. The leptin-to-adiponectin ratio was calculated as the circulating levels of leptin divided by circulating adiponectin levels. The adiponectin-to-leptin ratio was calculated as the circulating levels of adiponectin divided by circulating leptin levels. Different superscript lowercase letters (a, b) indicate significantly different mean values (one-way ANOVA and Duncan’s *post hoc* test or Welch test and Games‒Howell *post hoc* test, p < 0.05).

Acyc-Ma, acyclic middle-aged rats. CRP, C-reactive protein. HOMA-IR, homeostasis model assessment-estimated insulin resistance. Irreg-Ma, irregular middle-aged rats. MWAT, mesenteric white adipose tissue. NEFAs, nonesterified fatty acids. POWAT, periovarian white adipose tissue. Reg-Ma, regular middle-aged rats. Reg-Yng, regular young rats. R-QUICKI, revised quantitative insulin sensitivity check index. TC, total cholesterol. TG, triglycerides.

### Natural reproductive senescence produced slight changes in biometric parameters and classical glucose, lipid, and inflammation-related biomarkers

As expected, middle-aged rats displayed higher body weight at baseline than young rats (Table 1). For this reason, all the tissue weights are presented in Table 1 as relative weights (expressed as a percentage of the total body weight). To complement the relative weight data presented in Table 1, absolute tissue weights for all groups have been included in Supplementary Data S1. At the end of the study, the Reg-Ma and the Irreg-Ma rats showed higher body weight than the Acyc-Ma animals, whereas Reg-Ma displayed decreased relative liver weight than their Irreg-Ma and Acyc-Ma counterparts Table 1. This latter effect could be attributed, at least in part, to the slight differences in body weight reported among the three middle-aged groups of rats because the visual examination of the livers did not reveal apparent lipid accumulation in any of the groups. The Acyc-Ma group displayed a significant increase in the serum total cholesterol compared with their Reg-Ma counterparts (Table 1). No significant changes were found among the four groups in the circulating TG levels or the glucose and insulin homeostasis parameters. However, Acyc-Ma animals displayed numerically higher insulin, TG, and HOMA-IR values than their counterparts (Table 1). Similarly, no significant differences among groups were found in the circulating levels of the acute inflammation marker CRP (Table 1).

The Reg-Ma group exhibited higher adiponectin serum levels than the Acyc-Ma group, while no significant differences were found among the other groups (Table 1). Regarding leptin levels, although no significant differences were observed within the middle-aged animals, Reg-Ma and Irreg-Ma rats exhibited higher levels than the Reg-Yng group (Table 1). Despite these slight effects observed among groups in the circulating levels of these two key adipocytokines, all middle-aged groups exhibited a lower adiponectin/leptin ratio and a higher leptin/adiponectin ratio than the young animals, which would suggest an increased cardiometabolic risk with aging regardless of the estrous cycle status of middle-aged rats (Table 1).

### Natural female reproductive aging altered the circulating levels of sex hormones

**Figure 1:**
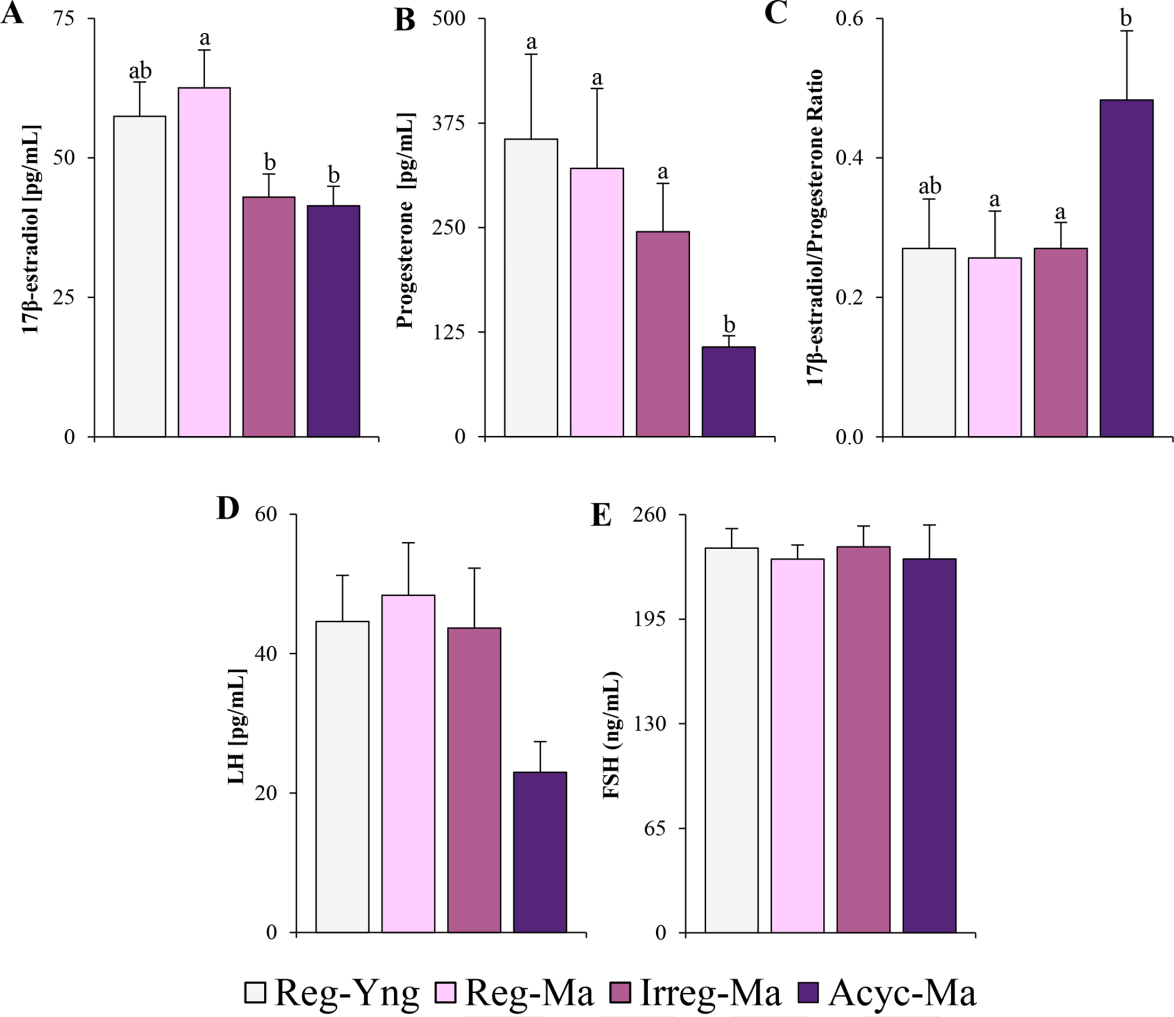
Circulating sex hormone levels during natural female reproductive aging in Sprague-Dawley rats. (**A**) Serum 17β-estradiol, (**B**) progesterone, (**C**) 17β-estradiol/progesterone ratio, (**D**) luteinizing hormone, and (**E**) follicle-stimulating hormone in ovarian-intact Sprague Dawley females at 5- and 10-month-old. The 17β-estradiol/progesterone ratio was calculated as the circulating levels of 17β-estradiol divided by circulating progesterone levels. Data are given as the mean ± SEM (*n* = 7–10). Different superscript lowercase letters (**a, b**) indicate significantly different mean values (one-way ANOVA and Duncan’s *post hoc* test or welch test and games‒Howell *post hoc* test, *P*<0.05). Reg-Yng, regular young rats; Reg-Ma, regular middle-aged rats; Irreg-Ma, irregular middle-aged rats; Acyc-Ma, acyclic middle-aged rats. LH, luteinizing hormone; FSH, follicle-stimulating hormone.

**Figure 2:**
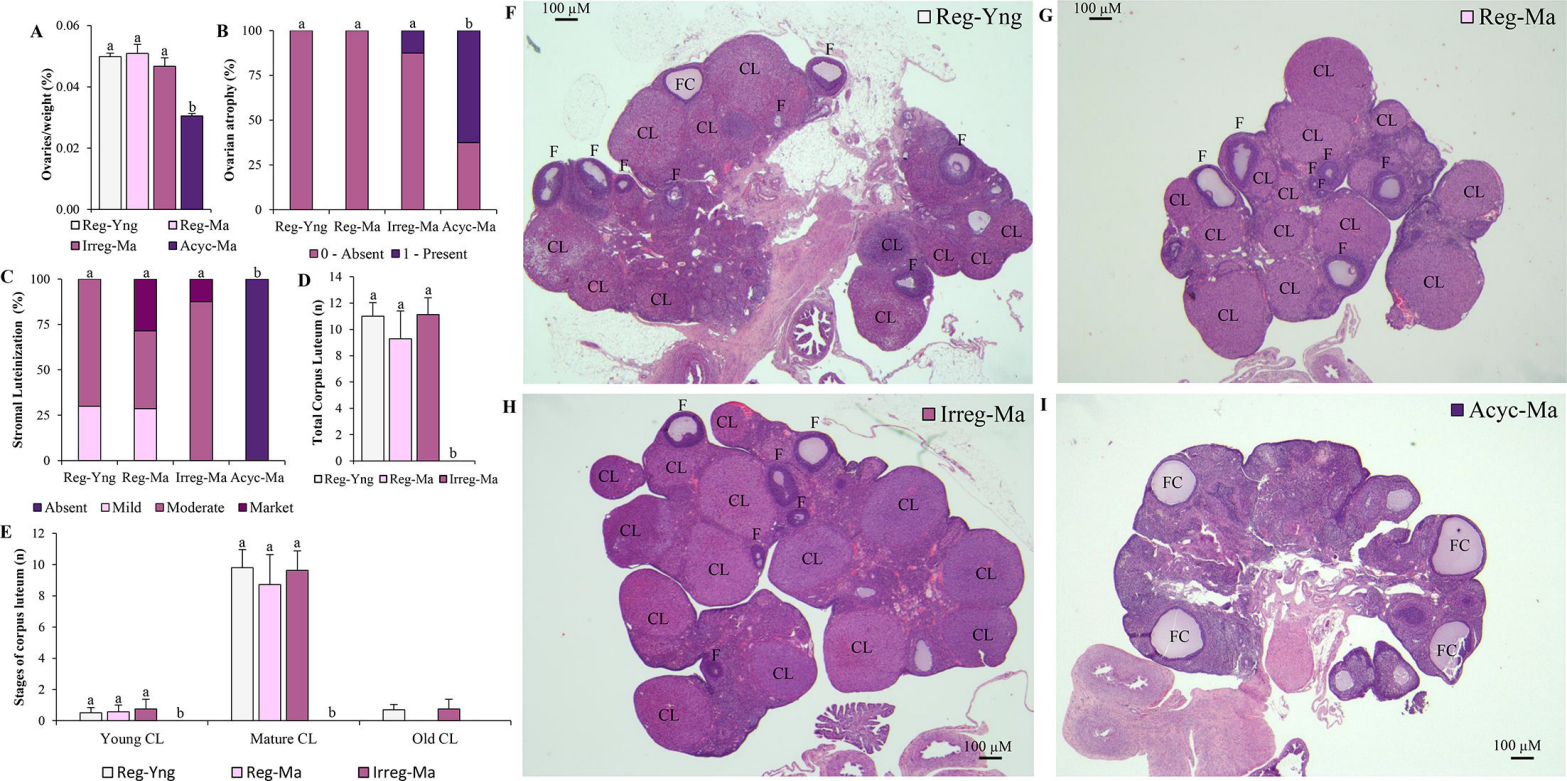
Ovaries/weight (%) and ovarian histological analyses. (**A**) Ovaries/weight(%), (**B**) ovarian atrophy (%), (**C**) stromal luteinization (%), (**D**) total number of Corpus Luteum (CL) and (**E**) stages of CL (n) in ovarian-intact Sprague Dawley females at 5- and 10-month-old. **Figures 2B and 2C** present qualitative observations, and different superscript lowercase letters (**a, b**) indicate significantly different mean values among groups (Pearson’s chi-square test, *P*<0.05). In **Figures 2A, 2D and 2E**, the data are given as the mean ± SEM (*n* = 7–10), and different superscript lowercase letters (**a, b**) indicate significantly different mean values among groups (Kruskal‒Wallis test and Mann‒Whitney U *post hoc* test, *P*<0.05). **Figures 2F-2I** represent the photomicrographs of hematoxylin–eosin-stained ovarian sections (Bar = 100 µM) in ovarian-intact Sprague Dawley females at 5- and 10-month-old. Reg-Yng, regular young rats; Reg-Ma, regular middle-aged rats; Irreg-Ma, irregular middle-aged rats; Acyc-Ma, acyclic middle-aged rats. CL, corpus luteum; F, secondaries, preantral and antral follicles; FC, follicles cysts.

**Figure 3:**
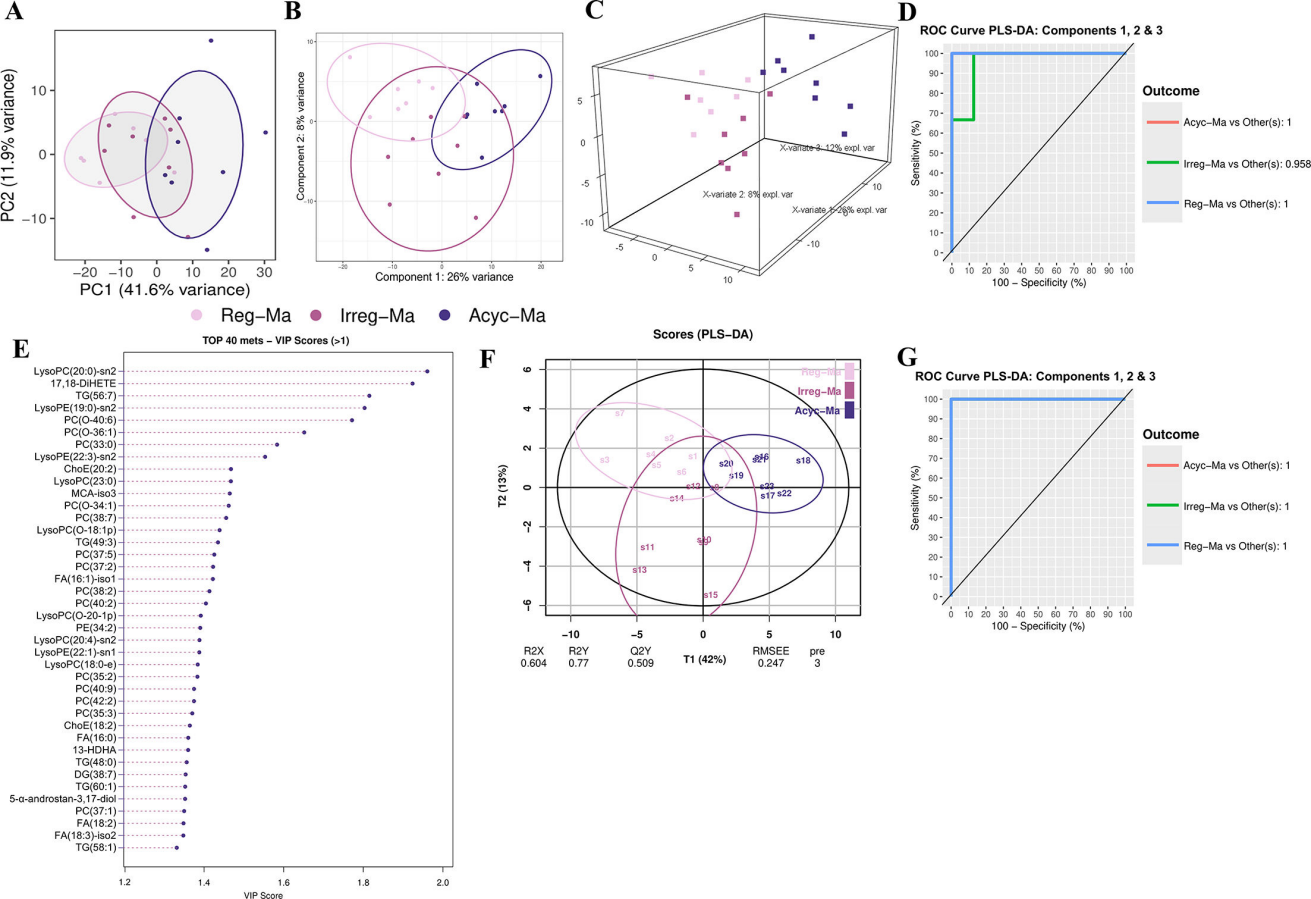
Effects of natural reproductive senescence on serum lipidomic of middle-aged female Sprague-Dawley rats. (**A**) Principal components analysis (PCA) plot of the 403 lipid metabolites. (**B**) PLS-DA predictive model based on the 403 lipid metabolites, with components 1 & 2. (**C**) 3D PLS-DA-predictive model, 3 components. (**D**) ROC curve of the 3-component PLS-DA model using the 403 lipid metabolites. (**E**) Distribution of lipid species with the top 40 VIP scores ( > 1) in the 3-component model. (**F**) The top 40 discriminant lipids with VIP scores > 1 were used to generate a predictive PLS-DA model with 3 components. (**G**) ROC curve of the 3-component PLS-DA model using the top 40 discriminant lipids. Reg-Ma, regular middle-aged rats; Irreg-Ma, irregular middle-aged rats; Acyc-Ma, acyclic middle-aged rats. PC1, principal component 1; PC2, principal component 2; PCA, principal components analysis; PLS-DA partial least square discriminant analysis; ROC curve, receiver operating characteristic curve; 5-α-androstan-3,17-diol, 5α-androstan-3,17-diol-monosulphate-iso3; 17,18-DiHETE, 17,18-dihydroxyarachidonic acid; ChoE, cholesterol ester; FA, fatty acids; 13-HDHA, 13-hydroxydocosahexaenoic acid; LysoPC, lysophosphatidylcholines; LysoPE, lysophosphatidylethanolamines; MCA-iso3, muricholic acid-iso3; PC, phosphatidylcholines; PE, phosphatidylethanolamines; TG, triglycerides.

17β-E2 circulating levels were similar between Reg-Yng and Reg-Ma animals and significantly decreased as mature female rats progressed to irregular cycles and reproductive senescence, showing both Irreg-Ma and Acyc-Ma animals similar values of this hormone (Figure 1A). As was previously described [12,14,15], progesterone serum concentrations were significantly decreased in the acyclic females (Figure 1B), an effect that could be attributed, at least in part, to a lack of ovulation and corpus luteum production. These effects were observed in the Acyc-Ma group according to the cytological analyses and the ovarian histological determinations carried out in these middle-aged animals (Figures 2 and 3). In addition, Acyc-Ma rats displayed a significant increase in the 17β-estradiol/progesterone ratio (Figure 1C), as was previously described [12,14] in animals presenting a persistent estrous state with an absence of corpus luteum. In this regard, a negative correlation was found between the 17β-estradiol/progesterone ratio and the total number of corpus luteum (*r* = -0.440, 95% CI = −0.114 to -0.681, *P*=0.010).

No significant changes among groups were observed in the circulating LH and FSH levels (*P*=0.099 and *P*=0.969, respectively, one-way ANOVA) (Figure 1D and E, respectively). However, subsequent pairwise comparisons revealed that serum LH concentrations residually decreased in acyclic rats when compared with their young and middle-aged regular counterparts (45.1% lower and 49.4%; *P*=0.029 and *P*=0.013, respectively, Student’s t-test), and a similar pattern of decrease was observed in acyclic versus irregular animals (43.9% lower, *P*=0.066, Student’s t-test) (Figure 1D).

### Gonadal-related parameters were altered across perimenopause

The Acyc-Ma animals displayed significantly lower relative weight of the ovaries than the other groups (Figure 2A), suggesting ovary atrophy, which was confirmed with the histological analyses, in which it was observed that 60% of the acyclic rats showed this alteration (Figure 2B). A very similar pattern was reported in the three groups of middle-aged rats regarding the POWAT/weight (%), displaying the Acyc-Ma animals decreased relative weight of the WAT surrounding the ovaries compared with their Reg-Ma and Irreg-Ma counterparts (Table 1). In contrast, the Acyc-Ma group showed higher uterine horns/weight (%) than the other groups, suggesting differences in uterine morphology associated with acyclic reproductive patterns (Table 1).

The ovarian histological analyses revealed that acyclic rats did not present either stromal luteinization or corpus luteum (Figure 2C and D), differing from what was observed in the other three groups of animals, which showed similar stromal luteinization (Figure 2C), and stages of development of the corpus luteum (Figure 2D), indicating that in the Reg-Yng, Reg-Ma, and Irreg-Ma groups, there was a progression of follicular luteinization towards the formation and development of normal corpus luteum during the estrous cycle. The lack of corpus luteum reported in the Acy-Ma rats is illustrated in the representative ovarian section depicted in Figure 2I, which showed the characteristic pattern of changes occurring in persistent estrus, including the presence of numerous follicular cysts (FC) and the absence of corpus luteum [12,19]. By contrast, the young cyclic rats (Figure 2F) and both the middle-aged cyclic and irregular animals (Figure 2G and H) exhibited the presence of follicles and corpus luteum in the ovary.

### Identification of lipidomic-based signatures across perimenopause

To identify changes in circulating metabolites occurring along natural reproductive senescence, semi-targeted metabolomic and lipidomic analyses were carried out in the serum of the Reg-Ma, Irreg-Ma, and Acyc-Ma groups. The initial unsupervised PCA performed on the polar metabolomics did not reveal separation trends among the groups. Furthermore, after adjusting p-values for FDR, no significant changes were found in the circulating levels of polar metabolites among groups (p adj>0.05, one-way ANOVA). More data are available in Supplementary data S2.

Regarding the semi-targeted serum lipidomic analyses, the initial unsupervised PCA performed with 403 hydrophobic metabolites revealed slight separation trends among the three groups at different stages of natural reproductive senescence (Figure 3A). A subsequent PLS-DA was conducted to explore the lipidomic differences among groups further. The PLS-DA plot (Figure 3B) and its three-dimensional representation with three components (Figure 3C) showed a clear separation and discrimination among the Reg-Ma, Irreg-Ma, and Acyc-Ma groups. The robustness of this model was validated through a permutation test with 999 iterations using a 5-fold cross-validation method, showing statistical significance (*P*=0.014) and a classification error rate (CER) of 0.397. Additionally, ROC curve analysis demonstrated an area under the curve (AUC) higher than 0.7 in all group comparisons (Figure 3D), supporting the model’s discriminatory performance.

Following the successful establishment of this PLS-DA model, VIP scores were calculated to identify the lipid-related metabolites that contributed more to discriminate the three groups. Based on VIP values with a threshold of 1, we selected the top 40 metabolites out of 186 lipids with VIP values higher than 1, including 14 phosphatidylcholines (PC), 6 lysophosphatidylcholines (LysoPC), 5 TG, 4 fatty acids (FA), 3 lysophosphatidylethanolamines (LysoPE), 2 cholesterol esters (ChoE), the phosphatidylethanolamine PE(34:2), the diacylglyceride DG(38:7), the androgen 5α-androstane-3,17-diol-monosulfate-iso3 (5-α-androstan-3,17-diol), the muricholic acid (MCA-iso3), 13-hydroxydocosahexaenoic acid (13-HDHA) and 17,18-Dihydroxyarachidonic acid (17,18-DiHETE) (Figure 3E). In addition, the top 40 discriminant lipids (VIP >1) were used to construct a PLS-DA predictive model with 3 components. The model achieved satisfactory performance metrics, including R²X = 0.604, R²Y = 0.770, and Q² = 0.509 (Figure 3F). Furthermore, the ROC curve based on these top 40 metabolites showed an AUC higher than 0.7 in all comparisons among groups (Figure 3G), demonstrating the utility of these lipid-related metabolites for discriminating rats according to their estrous cycle regularity. These results suggest that these 40 lipid-related metabolites could serve as reliable multivariate biomarkers to precisely distinguish rats at different stages of perimenopause (Figure 3E).

**Figure 4:**
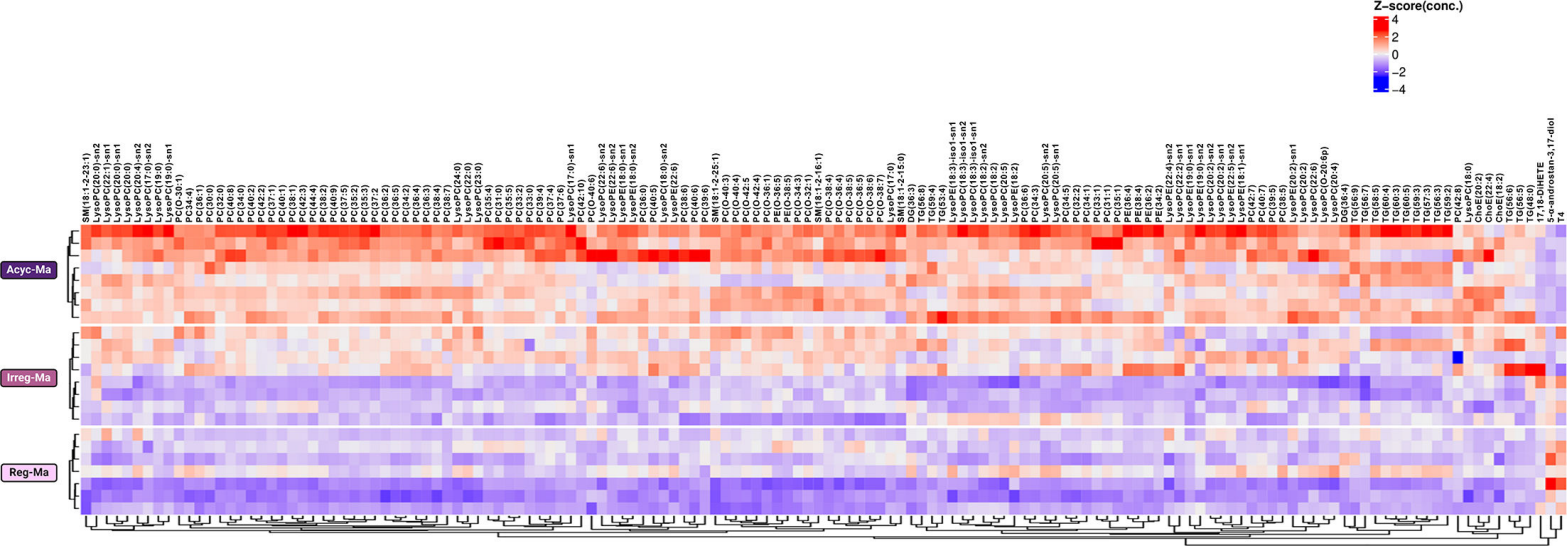
Effects of natural reproductive senescence on serum lipidomic of middle-aged female Sprague-Dawley rats. Heatmap visualizing (Z score) 144 significant lipids altered (adj. *P*<0.05). Reg-Ma, regular middle-aged rats; Irreg-Ma, irregular middle-aged rats; Acyc-Ma, acyclic middle-aged rats. 5-α-androstan-3,17-diol, 5α-androstan-3,17-diol-monosulphatemonosulfate-iso3; 17,18-DiHETE, 17,18-dihydroxyarachidonic acid; ChoE, cholesterol ester; DG, diacylglycerides; LysoPC, lysophosphatidylcholines; LysoPE, lysophosphatidylethanolamines; PC, phosphatidylcholines; PE, phosphatidylethanolamines; SM, sphingomyelins; T4, thyroxine hormone; TG, triglycerides.

On the other hand, the univariate statistical analysis revealed that 144 out of 403 hydrophobic metabolites identified were significantly different among Reg-Ma, Irreg-Ma, and Acyc-Ma groups (p adj<0.05, one-way ANOVA). The fold changes, statistical data, and raw data from this analysis are summarized in Supplementary data S3. Specifically, changes in the circulating levels of 68/76 PCs, 30/80 LysoPCs, 16/65 TGs, 12/45 LysoPEs, 6/7 phosphatidylethanolamines (PE), 4/16 ChoEs, 4/23 sphingomyelins (SM), 2/13 diacylglycerides (DG), 5-α-androstan-3,17-diol, thyroxine hormone (T4), and 17,18-DiHETE were found. Notably, the families of lysophospholipids showed the highest saturation levels, being saturated with 40% of LysoPCs and 33% of LysoPEs. In contrast, the families showing the lowest saturation levels were PCs (9%) and TGs (6%). We constructed a heatmap using the 144 lipids that showed significant changes to better illustrate the observed changes. The clustering by group regularity was evident, providing a more precise visualization of the lipid-related metabolite variations according to cycle regularity (Figure 4).

Interestingly, both the multivariate analysis of the top 40 metabolites with VIP scores higher than 1 and the univariate analysis, which showed that 144 lipids were significantly different under one-way ANOVA, revealed a higher proportion of PC and LysoPC families, the abundance of which differed among the Reg-Ma, Irreg-Ma, and Acyc-Ma groups. In addition to cholesterol esters ChoE(18:2), ChoE(20:2), the androgen 5-α-androstan-3,17-diol, (a derivative of dehydroepiandrosterone sulfate -DHEA-S- and dihydrotestosterone -DHT-), 17,18-DiHETE (a metabolite derived from arachidonic acid), TG(48:0) and TG(57:6), metabolites within these families were consistently identified after both analyses: PC(33:0), PC(35:2), PC(35:3), PC(37:1), PC(37:5), PC(38:2), PC(38:7), PC(40:2), PC(40:9), PC(42:2), PC(O-36:1), PC(O-40:6), LysoPC(20:0)-sn2, LysoPC(23:0), LysoPC(20:4)-sn2 and PE(34:2).

**Figure 5:**
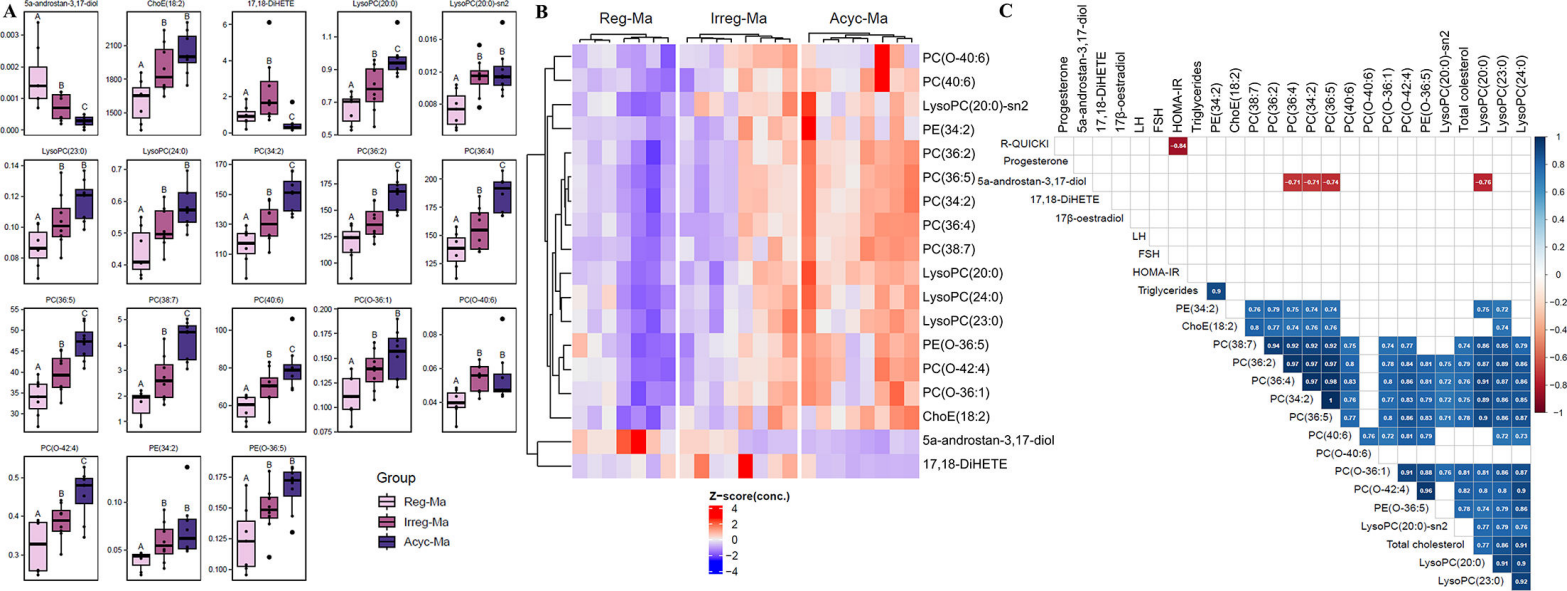
Effects of natural reproductive senescence on serum lipidomics of middle-aged female Sprague-Dawley rats. (**A**) Boxplots showing the 18 significant early lipid biomarkers selected by *P*<0.05 in post hoc test applied between regular and irregular middle-aged rats. Different superscript lowercase letters (**A, B, C**) indicate significantly different mean values (one-way ANOVA and Duncan’s post hoc test or Welch test and Games‒Howell post hoc test, *P*<0.05). (**B**) Heatmap visualizing (Z score) top 18 significant lipids altered (adj. *P*<0.05) between regular and irregular middle-aged rats. (**C**) Bonferroni-adjusted Pearson correlation matrix of significant circulating hormone and metabolic levels and 18 significant metabolites. Only correlations adjusted with *P*<0.05 are plotted. Reg-Ma, regular middle-aged rats; Irreg-Ma, irregular middle-aged rats; Acyc-Ma, acyclic middle-aged rats. 5-α-androstan-3,17-diol, 5α-androstan-3,17-diol-monosulphate-iso3; 17,18-DiHETE, 17,18-dihydroxyarachidonic acid; ChoE, cholesterol ester; FSH, follicle-stimulating hormone; HOMA-IR, homeostasis model assessment-estimated insulin resistance; LH, luteinizing hormone; LysoPC, lysophosphatidylcholines; PC, phosphatidylcholines; PE, phosphatidylethanolamine.

To detect putative early biomarkers of natural female reproductive aging, we selected, among the 144 metabolites that were significantly different among the three groups using the one-way ANOVA, those lipid-related metabolites that were significantly different between Reg-Ma and Irreg-Ma rats, according to the Duncan *post hoc* test. In total, we identified 18 parameters that already changed with the transition from the regular estrous cycle characteristic of the Reg-Ma animals to the irregular cycling observed in the Irreg-Ma rats, suggesting that these metabolites could be potential early biomarkers of perimenopause. Specifically, we found changes in the circulating levels of 9 PCs, 4 LysoPCs, 2 PEs, ChoE(18:2), 5-α-androstan-3,17-diol, and 17,18-DiHETE. A boxplot including these 18 metabolites and their relative quantification in the three middle-aged groups was depicted in Figure 5A. A consistent trend of increasing values was observed as the cyclicality diminished for most of these parameters, except 5-α-androstan-3,17-diol, which displayed an inverse behavior. Finally, we generated a new heatmap specifically focusing on these 18 metabolites, clearly illustrating their discriminatory pattern between the Ma-Reg and the Irreg-Ma groups (Figure 5B). A Bonferroni-adjusted Pearson correlation matrix plot was generated to visualize the relationships between these 18 metabolites and the circulating levels of the main sex hormones, triglycerides, and total cholesterol, as well as the surrogate markers of insulin resistance and sensitivity, HOMA-IR, and R-QUICKI, respectively (Figure 5C). Surprisingly, although no correlations were found with the analyzed sex hormones, the androgen derivative of DHT, 5-α-androstan-3,17-diol, showed strong negative correlations with the phosphatidylcholines PC(34:2), PC(36:4), PC(36:5), and LysoPC(20:0). Furthermore, as expected, these mentioned PC species exhibited robust positive correlations among themselves, as well as with the other PC species and with LysoPCs. This observation may be attributed to their metabolic interconversion, which supports a strong and positive correlation. Interestingly, total serum cholesterol positively correlated with most PC and LysoPC species, while serum triglycerides showed a strong positive correlation with PE(34:2).

### Bone-related parameters did not change during natural female reproductive aging

No significant changes were found among groups in bone-related parameters, including the weight and length of femurs, as well as the femur bone mineral content (BMC) and bone mineral density (BMD) analyzed by microCT (Table 2).

**Table 2:** Bone-related parameters in female rats during reproductive aging.

	Reg-Yng	Reg-Ma	Irreg-Ma	Acyc-Ma
Biometric parameters				
Femurs (g)	1.75 ± 0.03	1.85 ± 0.06	1.82 ± 0.03	1.79 ± 0.03
Femurs (cm)	3.55 ± 0.02	3.61 ± 0.03	3.64 ± 0.03	3.59 ± 0.03
Microcomputed tomography analyses				
BMD (mg/cc)	750 ± 10	709 ± 17	732 ± 17	749 ± 15
BMC (mg)	522 ± 12	547 ± 17	563 ± 5	557 ± 9

Data are given as the mean ± SEM (n = 7-8). Microcomputed tomography analyses in the femur were quantified from microCT scans using GE MicroView software v2.2.

Acyc-Ma, Acyclic middle-aged rats. BMC, bone mineral content. BMD, bone mineral density. Irreg-Ma, irregular middle-aged rats. Reg-Ma, regular middle-aged rats. Reg-Yng, regular young rats.

## Discussion

Reproductive senescence, resulting from hypothalamic and ovarian aging, manifests estrous cycle irregularities in rats and mice, marking the decline of their reproductive lifespan. Assessments of rodent estrous cyclicity can offer mechanism insights into potential adverse effects during the early menopausal transition, given the similarities in the regulatory mechanisms of the female reproductive axis across species [13,15,34]. Two studies have reported molecular neuroendocrine alterations in the hypothalamus affecting HPO functions, leading to irregular reproductive cycles and acyclicity in middle-aged female Sprague-Dawley rats similar to menopausal transition in women [18,23]. In one of these studies, Kermath *et al*. examined the circulating levels of sex hormones and found no significant differences in estradiol [23]. However, our study revealed lower levels of this hormone associated with the onset of cycle irregularity and acyclicity, which would resemble the situation occurring in women. In this regard, the SWAN study, which followed 3302 women aged 42–52 over 8 years, found that the circulating levels of estradiol remained stable until a rapid decline during the last two years of the late phase in the perimenopausal woman, with levels settled at low values two years after the final menstrual period (FMP) [35]. Furthermore, in women, the late menopausal transition is also characterized by an increase in the circulating levels of FSH [23,35,36] and, in agreement with this behavior, Kermath *et al*. reported a surge in FSH levels coinciding with the onset of irregularity and acyclicity in middle-aged rats [23]. By contrast, we did not observe such differences, which could be attributed at first glance to the fact that rats were not sacrificed during the estrus phase of the cycle, when the FSH secretion peak occurs [30]. Additionally, in our study, the decreased LH levels observed in the acyclic animals, when compared with their middle-aged regular and irregular counterparts would indicate altered gonadotropin secretion patterns during reproductive aging [37–39], a pattern that was also observed in Kermath *et al*., although the differences were not statistically significant [23]. While a regular vaginal cycle does not always indicate ovulation, stromal luteinization, which confirms ovulation, transforms follicles into corpus luteum [16]. As previously described in numerous studies, persistent estrus is characterized by low levels of progesterone and an increased 17β-estradiol/progesterone ratio, which is attributed to a lack of ovulation and corpus luteum production and is also accompanied by the presence of numerous follicular cysts and age-related ovarian atrophy [12,13,15,19,29,40]. In our study, all these hallmarks were reported in the acyclic group, reinforcing the translational applicability of this natural female reproductive aging rat model, characterized by a decline in ovarian function during reproductive senescence caused by hormonal imbalances and changes in the HPO axis, leading to ovulation failure and the end of the estrous cycle.

The hormonal and histological alterations detailed above can have profound implications beyond the reproductive system. Given that lipid compounds, such as glycerolipids, glycerophospholipids, and sterols, play critical roles in cellular structure, energy storage, and signaling, changes in the circulating lipidome can reflect and potentially drive changes in metabolic and physiological states linked with endocrine function [41]. Lipidomics, the large-scale study of pathways and networks of cellular lipids in biological systems, provides a comprehensive understanding of the diverse lipid species and their roles in health and diseases such as cardiovascular disease (CVD), MetS, neurodegenerative diseases, and different types of cancer [41]. In our study, we identified circulating lipidomic signatures of perimenopause, reporting that the progressive transition of estrous cycles encompassing regular, irregular, and acyclic stages that characterized the natural reproductive senescence was associated with marked changes in serum lipid-related metabolites, with an independent effect of age. As a general trend, most of the identified lipid families (PCs, LysoPCs, PEs, LysoPEs, ChoEs, and TGs) increased as reproductive aging was more evident.

To date, only two studies reported changes in circulating lipidomic profiles between premenopausal and postmenopausal women. However, none of these studies directly examined these biomarkers in the early stages of perimenopause or longitudinally during the menopausal transition [24,25]. Nogueira *et al*. showed significant increases in PCs in the postmenopausal state, with strong positive correlations between total cholesterol and low-density lipoprotein cholesterol (LDL-c) and PC(38:1) and PC(38:2) [24]. The significant increase observed in our study in the circulating levels of these PCs among the Reg-Ma, Irreg-Ma, and Acyc-Ma groups is consistent with these previous findings. Notably, in our research, PC(38:2) was one of the top 40 metabolites that contributed more to discriminate the three groups, which would suggest that this lipid species is already altered in an early stage of female reproductive aging as occurred with PC(34:2), PC(36:2), and PC(36:4), which in the present research were significantly higher in irregular than in regular rats and displayed strong positive correlations with the circulating levels of cholesterol. Elevated plasma levels of PC(34:2) and PC(36:2) have been associated with type 2 diabetes [42]. However, in our study, these PC changes cannot be linked to impaired glucose and insulin metabolism because no significant changes among middle-aged groups were found in the surrogate markers of insulin resistance and sensitivity, HOMA-IR and R-QUICKI, respectively, and no correlations were found between these both parameters and the circulating levels of PC(34:2) and PC(36:2). Remarkably, the increase in the circulating levels of LysoPE(22:6), LysoPE(22:5), and LysoPC(17:0) and the decrease in the plasma 5-α-androstan-3,17-diol concentrations found in our study during the natural transition to reproductive senescence are in line with the results obtained by Ke *et al*., which reported that these lipid species followed the same behavior in postmenopausal women compared with climacteric women [25]. These findings would reinforce our idea that changes in lipid-related metabolites are already occurring in the early stages of perimenopause.

The higher levels of PCs, PEs, LysoPCs, and LysoPEs species observed in postmenopausal women in the aforementioned studies may reflect hormonal changes during menopause affecting liver lipoprotein metabolism [43] and could be involved in the appearance of metabolic-related alterations. Thus, fasting blood phospholipid composition primarily reflects hepatic phospholipid synthesis [44], and it was shown that saturated phospholipids were positively associated with CVD risk [45]. Furthermore, LysoPCs, the most abundant lysophospholipid species found in human blood, have been suggested to promote atherosclerosis by eliciting an inflammatory response when they contain saturated acyl chains [46,47]. Steinberg et al. also demonstrated that LysoPCs were presented in higher concentrations in oxidized LDLs [47]. In our study, 40% of the LysoPCs that significantly changed among the three middle-aged rat groups contained a saturated acyl chain, including the LysoPC(20:0), LysoPC(20:0)-sn2, LysoPC(23:0), and LysoPC(24:0) species, which were 4 out of 18 lipids that were already altered coinciding with the onset of irregular cycles, exhibiting significant increases compared with the regular cycle group. According to the VIP score analysis, LysoPC(20:0)-sn2 emerged as the most discriminative parameter. The combination of fatty acids bound at the sn-1 and sn-2 positions largely determines the biophysical properties of cell membranes, which influence the activities of integral membrane proteins [48,49] and provide substrates for signaling processes [50,51]. Therefore, factors that ensure membrane phospholipid homeostasis are crucial for maintaining cell function, with the liver also playing a relevant role, being involved in extracellular lysophospholipids level regulation by modifying the expression of several enzymes [52]. Related to this, lecithin cholesterol acyltransferase (LCAT) cleaves fatty acids from the sn-2 position of PC, resulting in LysoPC primarily containing saturated fatty acids [53]. Therefore, it is plausible to speculate that an increased plasma LCAT activity may be one of the factors causing the increase in the serum levels of saturated species of LysoPC observed in this study. Other studies have reported that elevated LysoPC levels can be produced by increased phospholipase A2 (PLA2) activity, which hydrolyzes PC, influencing cell proliferation, invasion, and migration in various cancers [53–57] and is overexpressed in breast and ovarian cancer patients [58–61]. In the present study, the circulating levels of TC progressively increased as there was a loss of cyclicity in the middle-aged rats and, interestingly, in the Irreg-Ma group, the increase reported in the LysoPC(20:0), LysoPC(20:0)-sn2, LysoPC(23:0), and LysoPC(24:0) plasma levels preceded the rise in TC, which was only significantly reported in rats displaying acyclicity. These results would suggest these four LysoPCs as potential early biomarkers of hypercholesterolemia and would agree with the potential harmful effect on the cardiometabolic health of the LysoPCs containing a saturated fatty acyl chain. Further studies, such as those carried out with rats challenged with a hyperlipidemic and atherogenic diet and analyzing additional related parameters, such as PLA2 and LCAT activities, at different time points, would be of interest to provide more insights into the pathophysiological significance of the increased LysoPC(20:0), LysoPC(20:0)-sn2, LysoPC(23:0), and LysoPC(24:0) circulating levels concerning CVD and cancer risks in the framework of menopausal transition and postmenopause.

In women, many studies reported that, regardless of the menstrual cycle or reproductive status, glycerophospholipid species concentrations are generally lowest and stable during the late 30 s to mid-40s, slightly increasing during the mid-40s and showing a higher increase in the late 40 s and early 50 s. This rapid increase in glycerophospholipid levels observed in women with age can partly be explained by a drop in estrogen levels after menopause [62]. Thus, higher concentrations of PC, PE, and TAG were found in postmenopausal women compared with premenopausal women [45], PCs being the species showing the greatest tendency to increase [45,63,64]. In our study, the increased circulating levels of PC(40:6), PC(36:5), PC(36:2), and PC(34:2) observed in rats in response to the progressive loss of estrous cycle regularity are in line with previous findings observed in women [65–67]. Furthermore, it was reported that the proportion of PE(O-36:5) was significantly greater in women than in men [65]. This metabolite was detected in our study as an early biomarker of the transition between regular and irregular cycles. It has been reported that men display a higher risk for CVD early in life, while women have a higher risk later in life [68,69]. Both the sex and age-related differences in CVD risk have been linked to changes in glycerophospholipid metabolism [70]. It is well known that circulating lipoproteins are affected by female sex steroids and that primary changes are observed during the altered fluctuations in sex hormone levels during the menopausal transition [71–73]. Whether the differences in the circulating glycerophospholipid composition reported in this rat model of natural female reproductive senescence could affect patterns of health and disease during perimenopause and contribute to sexual dimorphism in CVD risk at middle age deserves further research.

The progressive significant decrease reported in the circulating levels of 5-α-androstan-3,17-diol, a metabolite of DHEA-S and DHT linked to androgenic activity, as the loss of regularity occurred, would also contribute to reinforce the reliability and the translational applicability of our findings because it was observed that the circulating levels of DHT decreased along with the estradiol and testosterone concentrations in postmenopausal women compared with premenopausal women [74]. Similarly, a pronounced decline in DHEA and its sulfate form, DHEA-S, was described in aged women [75]. Interestingly, higher levels of PCs were linked to lower androgenic steroid levels in postmenopausal women [25,76]. In line with these results, our study found strong negative correlations between these metabolites and the phosphatidylcholines PC(34:2), PC(36:4), and PC(36:5). These findings suggest a link between androgenic hormones and PCs in reproductive senescence, although additional research would be needed to gain knowledge on this issue.

DiHETE family is associated with type II diabetes and inflammatory diseases [77,78]. The transitory increase reported in rats displaying irregular cycles concerning the circulating levels of 17,18-dihydroxyeicosatrienoic acid (17,18-DiHETE), a metabolite derived from the omega-6 fatty acid arachidonic acid, and, to a lesser extent, in the plasma concentrations of 13-HDHA, a metabolite derived from the omega-3 fatty acid docosahexaenoic acid (DHA) that was identified as one of the 40 metabolites that contributed more to discriminate the three groups of middle-aged rats, was, at first glance, an unexpected result. Intriguingly, the levels of these metabolites declined in the acyclic group of rats, showing these animals even lower levels than those reported in the middle-aged rats showing regular cycles. PC species stand as a primary reservoir of polyunsaturated fatty acids, such as arachidonic and linoleic acids. These fatty acids serve as precursors for eicosanoids, which exhibit diverse biological activities [79–81]. Interestingly, all the PCs that differed statistically between rats with regular and irregular cycles were polyunsaturated, except for PC(0–36:1). It is well-established that PUFA metabolites, including those originating from arachidonic acid, along with eicosapentaenoic acid (EPA) and DHA, play a key role in the regulation of the inflammatory response [82]. Estrogens stimulate DHA production in rats, suggesting post-menopause estrogen changes may affect DHA metabolism [83]. However, because the circulating levels of this hormone decreased at similar levels in both irregular and acyclic groups, it seems unlikely that estradiol could be responsible, at least in part, for the transitory effects observed in the circulating levels of 17,18-DiHETE and 13-HDHA. Further studies would be needed to clarify this issue.

Given that the average menopausal age for European women is 51 [84], the significant increases observed around age 50 in the above-commented lipid-related metabolites likely would correspond to changes in the reproductive status [71]. However, many studies overlook the fact that menopause is preceded by the menopausal transition, a period marked by menstrual cycle irregularity starting typically in the mid-40s [63], which could account for early changes in metabolites that could be useful as potential early biomarkers of perimenopause. In this regard, to the best of our knowledge, there are only two studies that reported changes in classical lipid-related metabolites within the 1 year interval before and after the FMP or during a follow-up carried out after 2.5 years during the menopausal transition, including increased concentrations of VLDLs, LDLs, total cholesterol, and LDL-cholesterol, apolipoprotein B, and reduced LDL particle size. These changes were independent of the women’s chronological age and may underlie women’s increased cardiometabolic risk in their post-menopausal years [71,72]. These studies highlight the importance of identifying biomarkers for early diagnosis of perimenopause to minimize the risk of suffering from CVDs. Our findings give additional insights into this issue, identifying up to 18 lipid-related metabolites that already changed with the appearance of the irregular cycles, and before that, a significant increase in the circulating levels of TC was reported. Therefore, it is tempting to speculate that these lipid-related compounds, including 9 PCs, 4 LysoPCs, 2 PEs, ChoE(18:2), 5-α-androstan-3,17-diol and 17,18-DiHETE, could be used as a potential multivariate biomarker to monitor, at molecular level, the response to new treatments and lifestyle-based interventions carried out at very early stages of natural reproductive senescence to preserve the endocrine and metabolic functions of perimenopausal women.

The present study has some limitations. First, the metabolites detected in this work were semi-quantified in terms of internal standard response ratio, and an absolute quantification by correlating signal intensities of the lipid-related metabolites with a calibration curve would be needed to validate the lipidomic changes reported during the perimenopausal transition. In addition, further advances in lipidomics platforms might help capture more comprehensive and complete lipidome profiles, including the position and saturation and/or unsaturation of fatty acyl chains in different lipid species, which can affect the functionality of lipid-related metabolites. Finally, our results were obtained at the preclinical level and with a relatively small sample size, and further clinical trials carried out with perimenopausal women would be needed to validate our findings. A thorough assessment of premenopause, perimenopause, and menopause would require highly detailed targeting of the final menstrual period and the appearance of preceding menopause-related symptoms, including longitudinal hormone and risk factor measures before, during, and after the menopausal transition. Incorporating lipidomic analysis into this assessment can provide deeper insights into the molecular changes occurring during these stages.

In conclusion, we showed that, in middle-aged rats, the natural menopausal transition, characterized by the gradual loss of regularity of the estrous cycle, led to significant progressive changes in the serum lipidomic profile, identifying circulating signatures of this biological process, including changes in the levels of lipids of the PCs, LysoPCs, PEs, LysoPEs, TGs, FAs, and ChoEs families. Remarkably, we identified a set of metabolites that already changed with the transition from a regular to an irregular estrous cycle, including 9 PCs, 4 LysoPCs, 2 PEs, ChoE(18:2), 5-α-androstan-3,17-diol, and 17,18-DiHETE, which could be used as a potential multivariate biomarker of early perimenopause. As far as we know, this is the first work reporting that lipidomic-related shifts in serum are already detected at very early stages of female reproductive senescence in a rat model that resembles the early menopausal transition occurring in women. Further clinical trials performed with this target population would be of great value in reinforcing our findings. A detailed understanding of the molecular changes occurring during the menopausal transition can pave the way to design new treatments and lifestyle-based interventions addressed to preserve the endocrine and metabolic functions of perimenopausal women and to ameliorate in later stages the vasomotor symptoms, osteoporosis, and metabolic-related diseases that can appear in postmenopausal women.

**Clinical perspectives**

- Perimenopause is a transitional phase marking the onset of irregular menstrual cycles and female reproductive senescence, which is characterized by declined ovarian function and altered sex hormone secretion and can be accompanied by health alterations. There is still a limited understanding of the underlying mechanisms associated with menopause transition, and filling this gap can pave the way for designing novel treatments aimed at improving the well-being and the health of both perimenopausal and postmenopausal women.
- Using a lipidomic approach we identified circulating signatures of perimenopause in an ovarian-intact middle-aged rat model mimicking this biological phase, detecting progressive changes in 144 serum metabolites among age-matched rats resembling pre-menopause (regular cycles), perimenopause (irregular cycles) and early postmenopause (acyclicity), including PCs, LysoPCs, PEs, LysoPEs, TGs, FAs, and ChoEs. Eighteen out of these 144 metabolites, including 9 PCs, 4 LysoPCs, 2 PEs, ChoE(18:2), 5-α-androstan-3,17-diol and 17,18-DiHETE already changed with the transition from regular to irregular cycles and anticipated the changes in blood progesterone, LH, and cholesterol levels occurring in acyclic rats. These metabolites could be used as a potential multivariate biomarker of early perimenopause.
- Although further clinical trials would be needed to validate our results, understanding the molecular changes occurring during menopausal transition could be of great value to develop targeted interventions aimed at preserving endocrine and metabolic functions in perimenopausal women and to ameliorate vasomotor symptoms, the osteoporosis and the metabolic risks associated with menopause throughout the remaining lifespan.

## Supplementary material

Online supplementary table 1

Online supplementary table 2

1undefined

2undefined

3undefined

## Data Availability

The original data supporting the findings of this study are available from the corresponding author upon reasonable request.

## Abbreviations

ANOVA: analysis of variance
AUC: area under the curve
Acyc-Ma: acyclic middle-aged rats
BMC: bone mineral content
BMD: bone mineral density
CRP: C-reactive protein
CVDs: cardiovascular diseases
ChoE: cholesterol ester
DG: diacylglycerides
DHEA-S: dehydroepiandrosterone sulfate
DHT: dihydrotestosterone
17,18-DiHETE: 17,18-dihydroxyarachidonic acid
ESI: electrospray ionization
FA: fatty acid
FDR: false discovery rate
FMP: final menstrual period
FSH: follicle-stimulating hormone
GC: gas chromatography
13-HDHA: 13-hydroxydocosahexaenoic acid
HOMA-IR: homeostasis model assessment-estimated insulin resistance
HPO: hypothalamic-pituitary-ovarian
Irreg-Ma: irregular cycling middle-aged rats
LDL: low-density lipoprotein
LH: luteinizing hormone
LysoPC: lysophosphatidylcholine
LysoPE: lysophosphatidylethanolamine
MCA-iso3: muricholic acid-iso3
MWAT: mesenteric white adipose tissue
MetS: metabolic syndrome
PC: phosphatidylcholine
PCA: principal components analysis
PE: phosphatidylethanolamine
PLS-DA: partial least-squares discriminant analysis
POWAT: periovarian white adipose tissue
QTOF: quadrupole time-of-flight
ROC: receiver operating characteristic
R-QUICKI: revised quantitative insulin sensitivity check index
Reg-Ma: regular cycling middle-aged rats
Reg-Yng: regular cycling young rats
SM: sphingomyelins
T4: thyroxine hormone
TC: total cholesterol
TG: triglycerides
UHPLC: ultra-high-performance liquid chromatography
VIP: variable importance in projection
VLDL: very low-density lipoprotein
5-α-androstan-3,17-diol: 5α-androstane-3,17-diol-monosulfate-iso3
17β-E2: 17β-estradiol
