# Supplementary material for: Impact of the natural female reproductive aging on the rat serum lipidome
Source: Clin Sci (Lond). 2025 Sep 18;139(18):957–77. doi: 10.1042/CS20255841 (PMC12616427; doi:10.1042/CS20255841)
Supplement: Online supplementary table 1 [file cs-139-18-CS20255841-s001.docx]

| Supplementary Table 1. UHPLC gradient | | |  |  | | |  |  |
| --- | --- | --- | --- | --- | --- | --- | --- | --- |
| **ESI (-)** | | |  | **ESI (+)** | | |  |  |
| **Time (min)** | **%B** | **Flow (mL/min)** |  | **Time (min)** | **%B** | **%C** | **%D** | **Flow (mL/min)** |
| **0** | 2 | 0.4 |  | **0** | 10.0 | 45.0 | 5.0 | 0.6 |
| **1.5** | 2 | 0.4 |  | **0.5** | 10.0 | 45.0 | 5.0 | 0.6 |
| **2.25** | 7.5 | 0.4 |  | **1.5** | 9.5 | 47.7 | 5.0 | 0.6 |
| **8.0** | 30 | 0.4 |  | **1.6** | 7.5 | 58.5 | 5.0 | 0.6 |
| **11.0** | 50 | 0.4 |  | **5.0** | 7.0 | 61.2 | 5.0 | 0.6 |
| **25.0** | 85 | 0.4 |  | **5.1** | 4.0 | 77.4 | 5.0 | 0.6 |
| **26.0** | 95 | 0.4 |  | **7.5** | 3.5 | 80.1 | 5.0 | 0.6 |
| **30.0** | 95 | 0.4 |  | **9.0** | 3.5 | 80.1 | 5.0 | 0.6 |
| **30.1** | 2 | 0.4 |  | **9.5** | 0.0 | 100.0 | 0.0 | 0.6 |
| **31.0** | 2 | 0.4 |  | **11.5** | 0.0 | 100.0 | 0.0 | 0.6 |
| **34.0** | 2 | 0.4 |  | **11.6** | 10.0 | 45.0 | 5.0 | 0.6 |
|  |  |  |  | **14.0** | 10.0 | 45.0 | 5.0 | 0.6 |
