# Supplementary material for: Impact of the natural female reproductive aging on the rat serum lipidome
Source: Clin Sci (Lond). 2025 Sep 18;139(18):957–77. doi: 10.1042/CS20255841 (PMC12616427; doi:10.1042/CS20255841)
Supplement: Online supplementary table 2 [file cs-139-18-CS20255841-s002.docx]

| Supplementary Table 2. Source parameters | |
| --- | --- |
| **Parameter** | **ESI (+ & -)** |
| Gas Temp. (ºC) | 225 |
| Gas Flow (L/min) | 11 |
| Sheath Gas Temp. (ºC) | 300 |
| Sheath Gas Flow (L/min) | 12 |
| Nebulizer (psi) | 35 |
| Capillary Voltage (V) | 3500 |
| Nozzle Voltage (V) | 500 |
